# Trends in commonly used and potentially inappropriate medications in older Korean patients with polypharmacy

**DOI:** 10.1186/s12877-024-05141-8

**Published:** 2024-06-21

**Authors:** Woo-young Shin, Tae-Hwa Go, Jung-ha Kim

**Affiliations:** 1 Department of Family Medicine, Chung-Ang University College of Medicine, 102 Heukseok-ro, Dongjak-gu, Seoul, 06973 Republic of Korea; 2 Department of Biostatistics, Yonsei University Wonju College of Medicine, Wonju, Republic of Korea

**Keywords:** Polypharmacy, Aged, South Korea, Potentially inappropriate medication

## Abstract

**Background:**

Polypharmacy is a global public health concern. This study aimed to determine the prevalence of polypharmacy and trends in the use of commonly used and potentially inappropriate medications among older Korean patients.

**Methods:**

Individuals aged ≥ 65 years who were prescribed any medication between 2014 and 2018 were selected from the Korean National Health Information Database. Joinpoint regression analyses were used to determine trends in the age-adjusted polypharmacy rates by age group. The prescription rates of the most commonly used medications and the most commonly used potentially inappropriate medications were analysed by year or age group for patients with polypharmacy using the chi-square and proportion difference tests.

**Results:**

This study included 1,849,968 patients, 661,206 (35.7%) of whom had polypharmacy. Age-adjusted polypharmacy rates increased significantly between 2014 and 2018 (*P* = 0.046). Among patients with polypharmacy, the most commonly prescribed medications were aspirin (100 mg), atorvastatin, metformin, glimepiride, and rosuvastatin. The most commonly prescribed and potentially inappropriate medications were alprazolam, diazepam, amitriptyline, zolpidem, and dimenhydrinate. There was a significant decrease in the prescription rates for each of these drugs in 2018 compared with 2014 among patients with polypharmacy (all *P* < 0.001), whereas there was a significant increase in alprazolam prescription among patients aged ≥ 85 years when analysed by age group (*P* < 0.001).

**Conclusions:**

This study revealed an increasing prevalence of polypharmacy among older adults. Additionally, it highlighted that the utilisation of commonly prescribed potentially inappropriate medications, such as benzodiazepines and tricyclic antidepressants, has remained persistent, particularly among patients aged ≥ 85 years who practiced polypharmacy. These findings provide evidence-based guidance for the development of robust polypharmacy management strategies to ensure medication safety among older adults.

**Supplementary Information:**

The online version contains supplementary material available at 10.1186/s12877-024-05141-8.

## Background

Polypharmacy is emerging as a significant global public health concern in the context of ageing populations and the increasing prevalence of multimorbidity [1]. It is generally defined as the concurrent use of five or more medications and is associated with an increased risk of potentially inappropriate medication use, adverse drug reactions, hospitalisation, and mortality [2–5]. Importantly, it is well established that advanced age, residence in long-term care facilities, and multiple chronic conditions are associated with polypharmacy [2, 6]. Moreover, older adults are more vulnerable to drug toxicity and adverse events due to the pharmacokinetic and pharmacodynamic alterations associated with physiological ageing [7]. The World Health Organization has recently called on countries to emphasise medication safety and take proactive steps to reduce preventable medication-related harm [8]. Accordingly, there is a need to identify in-depth trends in polypharmacy in a large older population and the actual status of their harmful medication use as the global population ages and the healthcare environment continues to change. However, the predominant focus of polypharmacy studies has been on quantitative assessment, including prevalence and related health outcomes, in specific vulnerable populations [9–11].

Previous studies have shown an increase in the prevalence of potentially inappropriate medication use among older adults in various countries, including Korea, over the past two decades [12, 13]. A recent study estimated the prevalence of potentially inappropriate medications in older Korean outpatients to be as high as 81% [14]. Increased use of potentially inappropriate medications has also been associated with several adverse health outcomes, including falls, confusion, and mortality [15]. In a study conducted in Korea, Chae et al. showed that an increased number of prescriptions for potentially inappropriate medications was associated with a higher risk of emergency department visits or hospitalisations among older adults living in nursing homes [16]. Older adults practising polypharmacy are at a heightened risk of consuming potentially inappropriate medications and are more prone to experiencing medication-related harm compared to younger adults [5, 16]. Nevertheless, there has been a scarcity of comprehensive studies on trends in the utilisation of potentially inappropriate medications among older adults practising polypharmacy in Korea [17].

Therefore, the present study aimed to determine the prevalence of polypharmacy in older Korean patients over a duration of 5 years and identify the trends in the use of commonly used and potentially inappropriate medications among patients with polypharmacy.

## Methods

### Data source

This study used data from the National Health Information Database (NHID) of the Republic of Korea. It is based on the fee-for-service model of healthcare [18], and as part of the nation’s social security framework, virtually all Korean residents are obliged to join the national health insurance programme [19]. The NHID is a health insurance database hosted and overseen by the National Health Insurance Service. It encompasses a broad spectrum of data, comprising demographic details and medical treatment records from healthcare facilities across Korea [18, 19]. This public database was extracted, processed, and de-identified to facilitate its use for research purposes [18, 19].

Ethical standards were adhered to throughout the study in accordance with the principles outlined in the Declaration of Helsinki. Given the unique characteristics of the NHID, the Institutional Review Board of Chung-ang University Medical Center waived the requirement for written informed consent. Ethical approval for the study was obtained from the Institutional Review Board of the Chung-Ang University Medical Center (Approval No.: 2003-010-19308).

### Study patients

**Fig. 1:**
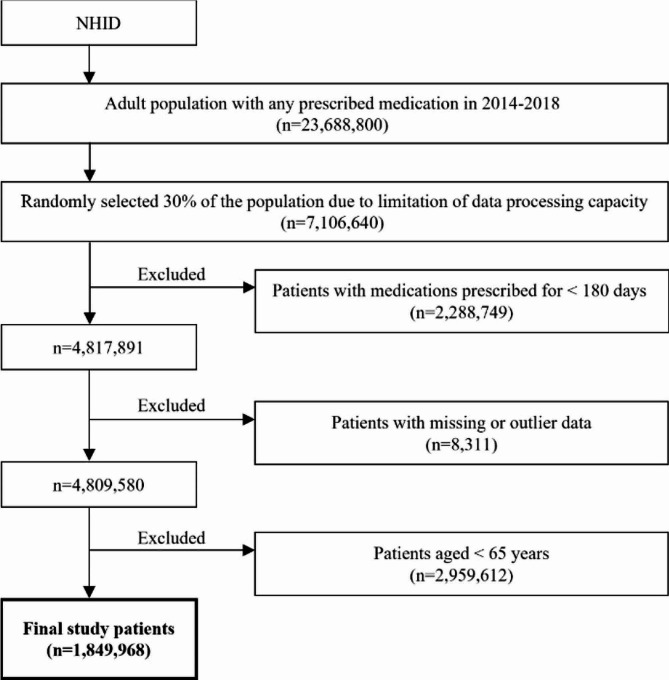
Flow chart of patient selection for this study

Adult populations who received at least one prescribed medication between January 1, 2014, and December 31, 2018, were chosen from the NHID. Roughly 30% of these data were randomly sampled for each year and included in the analysis. Patients who were prescribed medications for less than 180 days, as well as those with missing or incomplete demographic or prescription medication information were excluded from the study. Subsequently, among the remaining patients, only those aged 65 years and older were ultimately selected for the study (Fig. 1).

### Medication use

For each patient, we gathered information on the medications prescribed during the study period, including the main active ingredient, the number of medications, and the duration of time for which each medication was prescribed. Each medication was coded and analysed according to the main active ingredient. We only collected data on oral medications prescribed for at least 180 days to exclude transient or unclear drug use. Polypharmacy was defined as the concurrent use of five or more medications with distinct active ingredients. Potentially inappropriate medications were evaluated based on the 2015 version of the Beers Criteria published periodically by the American Geriatrics Society. The Beers Criteria refers to periodically updated and published medication lists that highlight potentially inappropriate medications for prescribing to older adults. They are one of the most widely utilised guidelines for ensuring geriatric medication safety worldwide [15]. Of the lists in the Beers Criteria, we used a list of medications that should be avoided by most older adults.

### Statistical analysis

The study entailed a retrospective cross-sectional analysis of patients who received medication prescriptions each year. The results are expressed as frequencies or percentages. To compare polypharmacy and prescription rates for each medication across different age groups, the study patients were categorised into the following three groups: 65–74 years, 75–84 years, and ≥ 85 years. Polypharmacy rates by age group were estimated from 2014 to 2018 and compared using the chi-square test. Furthermore, age-adjusted rates were calculated by standardising them based on the mid-year population of Korean residents registered in 2010 [20]. Joinpoint regression analyses for trends in age-adjusted polypharmacy rates were performed for five consecutive years. The prescription rates of the five most commonly used medications in patients with polypharmacy were evaluated and compared year-wise using the chi-square test. The prescription rates of the five most commonly used potentially inappropriate medications according to the Beers criteria were also analysed for patients with polypharmacy. The chi-square test was used to assess differences in the prescription rates of each medication by age group in each year of the study period. Additionally, the percentage differences and changes in the prescription rate of each medication between 2014 and 2018 were calculated and statistically confirmed using the proportional-difference test. All analyses were conducted using SAS version 9.4 (SAS Institute Inc., Cary, NC, USA) and Joinpoint Regression Programme version 4.9.0.0 (Statistical Research and Applications Branch, National Cancer Institute). Statistical significance was set at *P* < 0.05.

## Results

**Fig. 2:**
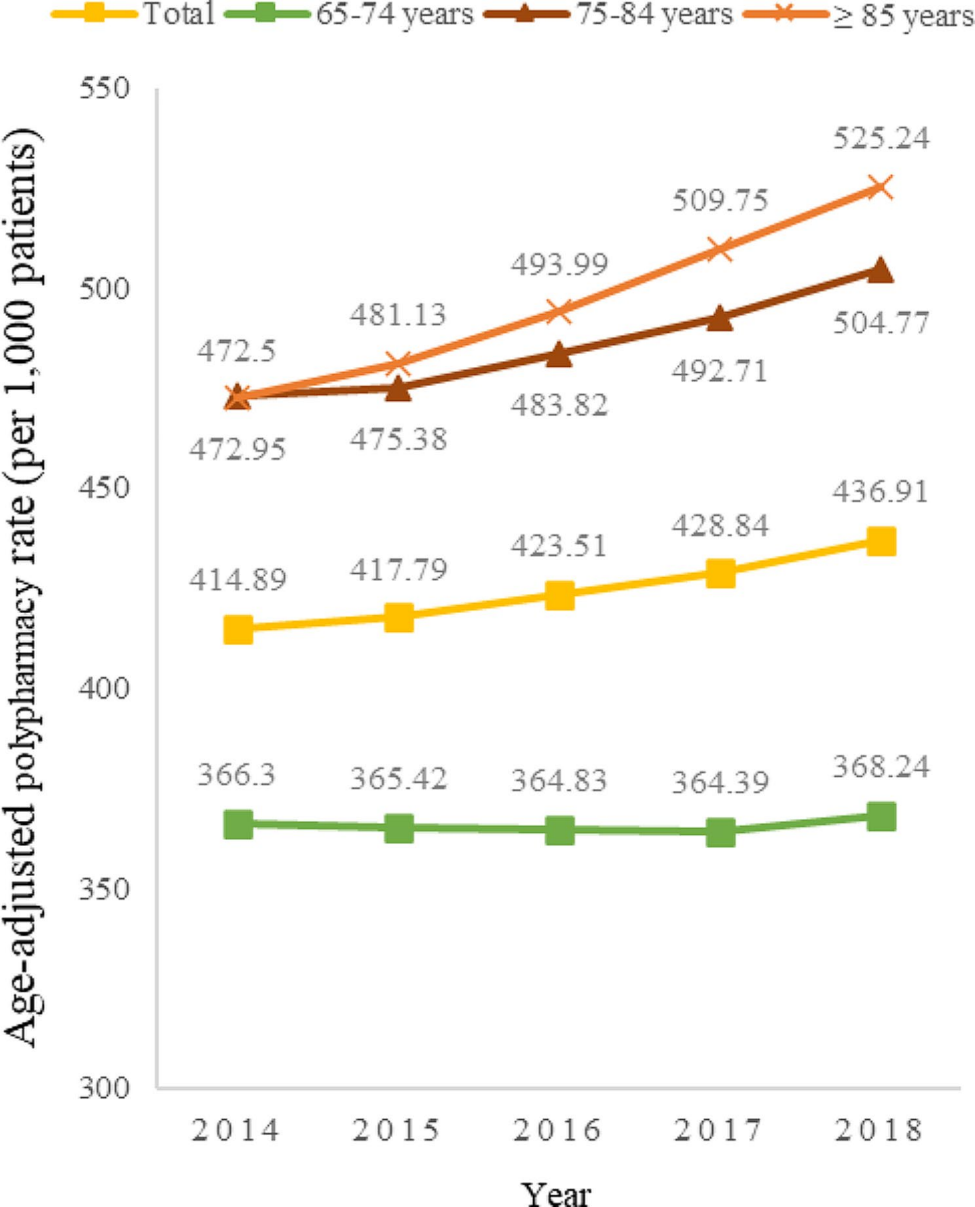
Trends in age-adjusted rates of polypharmacy in older Korean patients by age group, 2014–2018 (per 1,000 patients). The data were analysed using the Joinpoint regression method. The annual percentage change in this rate was statistically significant (*P* = 0.046)

A total of 1,849,968 patients prescribed any medication were included in the study (Fig. 1). Approximately 56% of these patients were aged 65–74 years, making them the largest age group. Of the total number of patients, 35.7% were reported to have polypharmacy during 2014–2018, with the highest rate of polypharmacy measured in the 75–84 age group at 42.0%. Polypharmacy rates were significantly different among the following age groups: 65–74 vs. 75–84 years, 65–74 vs. ≥85 years, and 65–74 vs. 75–84 vs. ≥85 years (all *P* < 0.001). However, polypharmacy rates in the 75–84 age group were not significantly different from those in the ≥ 85 age group (*P* = 0.453) (Table 1). Figure 2 shows the age-adjusted trends in polypharmacy rates per 1,000 patients by age group between 2014 and 2018. The annual percentage change in the age-adjusted polypharmacy rates was statistically significant (*P* = 0.046), and the rates steadily increased from 2014 to 2018. Polypharmacy rates have been the highest among patients aged ≥ 85 years since 2015 (Fig. 2).

**Table 1:** Age-wise usage of medications in older Korean patients in 2014–2018 (*n* = 1,849,968)^a^

Number of medications	65–74 years (*n* = 1,036,504)	75–84 years (*n* = 643,449)	≥ 85 years (*n* = 170,015)	Total (*n* = 1,849,968)
1	243,056 (23.5)	109,842 (17.1)	34,132 (20.1)	387,030 (20.9)
2	193,625 (18.7)	95,994 (14.9)	22,878 (13.5)	312,497 (16.9)
3	155,686 (15.0)	87,590 (13.6)	21,816 (12.8)	265,092 (14.3)
4	124,751 (12.0)	79,508 (12.4)	19,884 (11.7)	224,143 (12.1)
Polypharmacy^b^	319,386 (30.8)	270,515 (42.0)	71,305 (41.9)	661,206 (35.7)

^a^ Variables are expressed in terms of frequencies (percentages)

^b^ Polypharmacy was defined as the use of five or more medications. Polypharmacy rates were significantly different among the following age groups: 65–74 vs. 75–84 years, 65–74 vs. ≥85 years, and 65–74 vs. 75–84 vs. ≥85 years (all *P* < 0.001). Polypharmacy rates in the 75–84 age group were not significantly different from those in the ≥ 85 age group (*P* = 0.453)

Among the 661,206 patients with polypharmacy, the five most commonly prescribed drugs during the study period were aspirin (100 mg unit), atorvastatin, metformin, glimepiride, and rosuvastatin (Table 2). The prescription rates and annual trends for these drugs are listed in Table 2. The use of aspirin (*P* < 0.001), metformin (*P* = 0.001), and glimepiride (*P* < 0.001) decreased significantly from 2014 to 2018, whereas that of rosuvastatin increased (*P* = 0.028). The age-specific prescription rates for the most frequently utilised medications among patients with polypharmacy from 2014 to 2018 are detailed in Table S1. The five most commonly prescribed potentially inappropriate and avoidable medications in older adults during the study period were alprazolam, diazepam, amitriptyline, zolpidem, and dimenhydrinate in patients with polypharmacy (Table 3).

**Table 2:** Prescription rates of the five most commonly used medications in older Korean patients with polypharmacy, 2014–2018 (*n* = 661,206)

Number	Medication	Prescription rate (%)					*P*-value^a^
		2014	2015	2016	2017	2018	
1	Aspirin (100 mg unit)	39.91	38.09	36.17	34.47	32.63	< 0.001
2	Atorvastatin	26.62	26.52	26.89	26.82	26.98	0.072
3	Metformin	22.12	21.20	20.34	19.68	19.22	0.001
4	Glimepiride	15.65	14.67	13.74	12.97	12.19	< 0.001
5	Rosuvastatin	9.36	12.77	14.54	15.36	16.03	0.028

^a^ Analysed by the chi-square test

**Table 3:** Changes in prescription rates for the five most commonly used potentially inappropriate medications in older Korean patients with polypharmacy, 2014–2018 (*n* = 661,206)

Age group	Number	Medication^a^	Prescription rate (%)		Percentage difference	Percentage change	*P*-value^b^
			2014	2018			
Total	1	Alprazolam	11.52	10.29	-1.23	-10.68	< 0.001
	2	Diazepam	7.09	6.13	-0.96	-13.54	< 0.001
	3	Amitriptyline	6.77	5.71	-1.06	-15.66	< 0.001
	4	Zolpidem	6.21	5.45	-0.76	-12.24	< 0.001
	5	Dimenhydrinate	6.47	5.24	-1.23	-19.01	< 0.001
65–74 years	1	Alprazolam	10.62	8.85	-1.77	-16.67	< 0.001
	2	Diazepam	6.80	5.42	-1.38	-20.29	< 0.001
	3	Amitriptyline	6.89	5.37	-1.52	-22.06	< 0.001
	4	Zolpidem	5.55	4.81	-0.74	-13.33	< 0.001
	5	Dimenhydrinate	4.62	3.19	-1.43	-30.95	< 0.001
75–84 years	1	Alprazolam	12.48	11.07	-1.41	-11.30	< 0.001
	2	Diazepam	7.55	6.65	-0.90	-11.92	< 0.001
	3	Amitriptyline	6.87	5.99	-0.88	-12.81	< 0.001
	4	Zolpidem	6.66	5.54	-1.12	-16.82	< 0.001
	5	Dimenhydrinate	7.79	5.99	-1.80	-23.11	< 0.001
≥ 85 years	1	Alprazolam	11.79	12.37	0.58	4.92	< 0.001
	2	Diazepam	6.62	6.79	0.17	2.57	0.188
	3	Amitriptyline	5.83	5.90	0.07	1.20	0.553
	4	Zolpidem	7.48	7.18	-0.30	-4.01	0.019
	5	Dimenhydrinate	9.64	9.40	-0.24	-2.49	0.106

^a^ The medications analysed were based on the 2015 version of the American Geriatric Society Beers Criteria

^b^ Analysed by the proportion difference test

There was a significant decrease in the prescription rates for each of these drugs in 2018 (compared with 2014; all *P* < 0.001). However, when analysing subgroups by age, there was a significant increase in the prescriptions for alprazolam in patients aged ≥ 85 years (*P* < 0.001), whereas diazepam, amitriptyline, and dimenhydrinate showed no significant changes. Additional file 1 presents the trends in prescription rates for the five most commonly used potentially inappropriate medications among patients with polypharmacy by age group from 2014 to 2018. The prescription rates for each medication in each age group were significantly different every year (all *P* < 0.001) (Additional file 1).

## Discussion

In this study, we assessed the prevalence of polypharmacy in older Korean adults over a duration of 5 years and identified trends in the prescription of commonly used and potentially inappropriate medications.

Our study revealed that among older adults, the age-adjusted polypharmacy rate exhibited a consistent upward trend throughout the study period. Additionally, polypharmacy rates escalated with advancing age, with a particularly notable increase observed among those aged ≥ 85 years. Polypharmacy is becoming more prevalent in older adults, especially with increasing age. Importantly, age-related pharmacokinetic and pharmacodynamic changes make older patients more susceptible to drug-related harm [5, 7].

Our study focused on patients with polypharmacy and evaluated commonly prescribed or potentially inappropriate medications. We noted that even among older adults, polypharmacy rates increased with age, and the age groups with the highest prevalence of the top five most common potentially inappropriate medications tended to be older. For example, our findings showed that alprazolam and diazepam, two of the most commonly used potentially inappropriate medications in this study, were most commonly prescribed to patients aged 75–84 years in 2014. However, by 2018, they were most commonly prescribed to patients aged ≥ 85 years. Given that the global population is ageing and the rate of ageing is accelerating, our findings suggest that polypharmacy and potentially inappropriate medication use will continue to increase [21, 22].

The Beers Criteria have been periodically updated since 2011 to enhance clarity and incorporate the latest evidence and developments in the medical field. These updates involve the addition or removal of certain medications from the original list [21, 22]. As this study was based on the 2015 version of the Beers Criteria, aspirin and glimepiride were not included in the analysis as potentially inappropriate medications for older patients. We found that aspirin (100 mg) and glimepiride were among the most commonly used medications in older patients with polypharmacy, although their prescription rates gradually decreased. Aspirin (100 mg) has long been a common addition to prescriptions for the prevention of cardiovascular diseases. However, recent evidence suggests that aspirin increases the risk of major bleeding events, particularly in older adults, and its primary prevention in healthy adults has no clear benefit [21]. The 2023 update of the American Geriatrics Society Beers Criteria recommends avoiding the general use of aspirin for the primary prevention of cardiovascular diseases in older adults [21]. This underscores the importance of not only avoiding the routine initiation of aspirin treatment in most older patients but also considering deprescribing aspirin clinically when it is unnecessary. Glimepiride is a long-acting sulfonylurea antidiabetic that is associated with a high risk of severe hypoglycaemia [22]. Older patients are more susceptible to the risks associated with this medication as they exhibit slower metabolism and eliminate medications at a slower rate in general. Further, in these individuals, the typical hypoglycaemic symptoms may not be as pronounced or may be difficult to recognise [22]. Accordingly, glimepiride was added to the list of potentially inappropriate medications to avoid in most older adults in the 2019 update of the Beers Criteria [22]. Therefore, the findings from our study that they are among the top five most commonly prescribed medications in older patients with polypharmacy suggest that they should be considered in future approaches for managing medication safety.

Alprazolam and diazepam were potentially inappropriate medications, with the highest prescription rates in our study; importantly, both are benzodiazepines. In line with our results, some studies examining the prevalence of potentially inappropriate medications using the Beers Criteria in hospitalised older Chinese patients also found that benzodiazepines were the most commonly prescribed inappropriate medications [5, 23]. Older adults generally exhibit increased sensitivity to benzodiazepines, a phenomenon that exposes them to an increased risk of depression, cognitive impairment, delirium, falls, hospitalisation, and mortality [24]. However, the prevalence of benzodiazepine use increases with age, even among older adults [15]. In our study, zolpidem was identified as one of the top five most commonly prescribed and potentially inappropriate medications. As zolpidem, a hypnotic, acts as a benzodiazepine receptor agonist, it may increase the risk of adverse events such as delirium and falls, similar to benzodiazepines, in older adults [15]. According to our results, amitriptyline, a tricyclic antidepressant, and dimenhydrinate, a first-generation antihistamine, are among the most commonly used potentially inappropriate medications in patients with polypharmacy. They have strong anticholinergic activity and may cause unexpected and harmful effects in older adults, including sedation, confusion, constipation, and orthostatic hypotension [15]. Similar to our findings, a study conducted in Thailand reported first-generation antihistamines, including dimenhydrinates, to be among the top three potentially inappropriate medications [23]. A study conducted in South Korea reported that approximately 89% of the elderly patients in a long-term care hospital were prescribed medications with anticholinergic properties; individuals with more anticholinergic prescriptions had significantly higher polypharmacy rates and a greater number of potentially inappropriate medications [25]. The Beers Criteria were published to help guide prescription decisions for medications that are potentially inappropriate by weighing the benefits and harms in older patients [15]. However, in real-world clinical practice, it is necessary to consider various factors, including the patient’s condition, environment, and situation. For example, it may be necessary to use benzodiazepines for older patients with certain conditions, such as severe generalised anxiety disorder [21].

Interestingly, in this study, we found that while the proportion of older adults with polypharmacy increased, their prescription rates for the top five potentially inappropriate medications declined. This may reflect efforts to reduce the use of high-risk medications in older patients, as has been commonly reported. However, previous studies have reported an increase in the prevalence of potentially inappropriate medication use among older adults in various countries, including Korea, over the past two decades [12, 13]. Recent studies on potentially inappropriate medication use have reported psychotropics and antihistamines as the most frequent prescriptions, similar to our findings, but with different active ingredients [5, 23]. This suggests that our findings may merely reflect a decrease in the proportion of the top five medications in the total number of potentially inappropriate medications prescribed rather than a greater diversity in the types of inappropriate medications used. Further in-depth studies are required to confirm these findings. Nevertheless, our findings showed that alprazolam prescription increased in the group aged ≥ 85 years, while those of diazepam, amitriptyline, and dimenhydrinate did not significantly decrease.

This study suggests that public health policies addressing polypharmacy management should not solely aim to minimise the number of medications prescribed. Instead, adopting a qualitative approach to ensure appropriate medication use is crucial for achieving optimal medication safety among older adults. Deprescribing refers to the withdrawal of unsafe and clinically unnecessary medications for patients [23]. This can be recommended as an evidence-based strategy to effectively reduce exposure to medications that should be avoided in older adults [23]. It is necessary to provide healthcare providers with medication review and deprescribing guidelines developed for the Korean healthcare setting and to support their training. It is also necessary to ensure that appropriate evidence-based information is disseminated to patients, caregivers, and community residents. This study also provides meaningful evidence substantiating the urgent need for improvements in the healthcare system to prevent off-label or duplicate prescriptions of psychotropic medications for potentially high-risk patients. Initiatives to reduce potentially inappropriate medication use, especially among frail older adults living in long-term care facilities, should be prioritized. Considering that patients with polypharmacy usually visit multiple medical institutions in Korea, it may be an option to implement a primary care physician system for older patients [26].

This study had some limitations. The data analysed in this study were extracted from a database established for claims of healthcare services provided to patients and not for research purposes [18]. Notably, these compiled data lacked information on non-reimbursable prescription purchases and over-the-counter medications not covered by national health insurance. Nonetheless, as this study used a large nationwide sample, our results provide a meaningful clinical basis for improving future systems and guidelines for medication safety [18]. Second, medications that are potentially inappropriate for use in most older patients may be clinically appropriate in some circumstances; details of the individual patients’ clinical conditions were not available in this study. Third, our findings were derived from the Korean healthcare system; therefore, their generalisability to the global context may be limited. Local circumstances should be considered when interpreting and applying these findings to other populations.

## Conclusions

In conclusion, in this study, we identified that polypharmacy is on the rise in older adults and that the use of commonly prescribed potentially inappropriate medications, such as benzodiazepines and tricyclic antidepressants, has not decreased, especially in patients with polypharmacy aged 85 years and older. These findings underscore the significance of conducting medication reviews and implementing appropriate deprescribing practices, particularly for older patients experiencing polypharmacy. They also offer evidence-based guidance for formulating robust polypharmacy management strategies aimed at enhancing medication safety.

### Electronic supplementary material

Below is the link to the electronic supplementary material.

Supplementary Material 1

Supplementary Material 2

## Data Availability

Data are accessible from the National Health Insurance Service database. However, access to the data used in this study is only available to researchers upon approval. Further information is available on the National Health Insurance Sharing Service website (https://nhiss.nhis.or.kr).

## Abbreviations

NHID: National Health Information Database
