# Supplementary material for: Trends in commonly used and potentially inappropriate medications in older Korean patients with polypharmacy
Source: BMC Geriatr. 2024 Jun 21;24:542. doi: 10.1186/s12877-024-05141-8 (PMC11193228; doi:10.1186/s12877-024-05141-8)
Supplement: Supplementary file 1 — Supplementary Material 1 [file 12877_2024_5141_MOESM1_ESM.docx]

**Table S1.** Age-wise prescription rates for the five most commonly used medications in older Korean patients with polypharmacy, 2014–2018 (n=661,206).

| **Number** | **Medication** | **Prescription rate (%)** | | |
| --- | --- | --- | --- | --- |
|  |  | **65–74 years** | **75–84 years** | **≥85 years** |
| 1 | Aspirin (100 mg unit) | 52.32 | 51.21 | 45.43 |
| 2 | Atorvastatin | 43.44 | 37.61 | 25.68 |
| 3 | Metformin | 41.12 | 32.85 | 19.64 |
| 4 | Glimepiride | 26.18 | 21.04 | 12.72 |
| 5 | Rosuvastatin | 24.96 | 19.39 | 11.31 |
