# Supplementary material for: Trends in commonly used and potentially inappropriate medications in older Korean patients with polypharmacy
Source: BMC Geriatr. 2024 Jun 21;24:542. doi: 10.1186/s12877-024-05141-8 (PMC11193228; doi:10.1186/s12877-024-05141-8)
Supplement: Supplementary file 2 — Supplementary Material 2 [file 12877_2024_5141_MOESM2_ESM.docx]

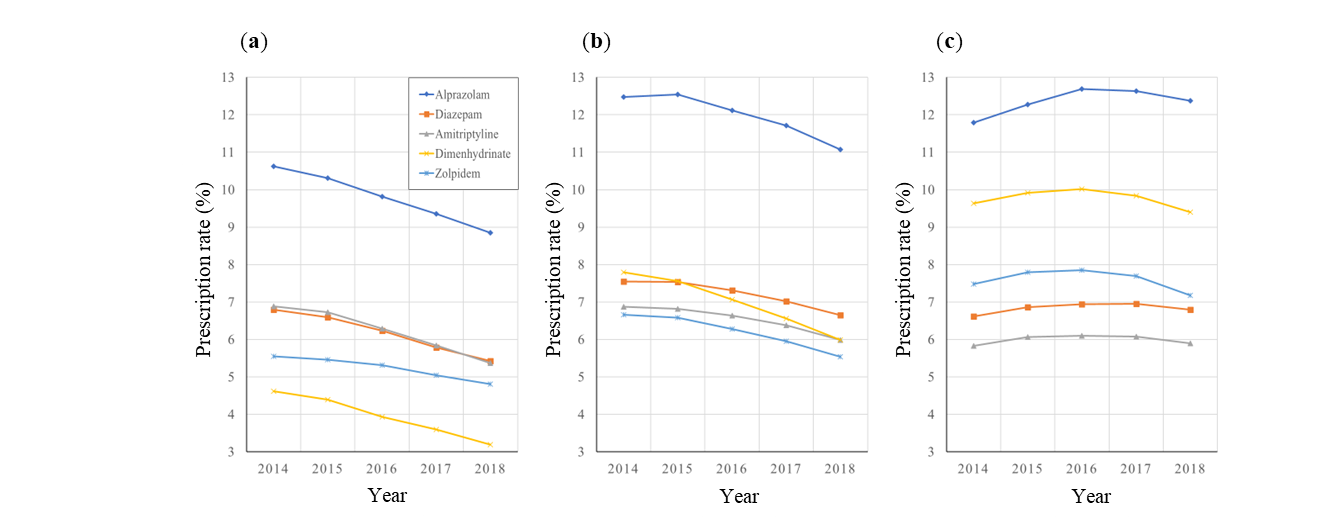


**Fig. S1.** Trends in prescription rates for the five most commonly used potentially inappropriate medications in older Korean patients with polypharmacy, 2014–2018 (n=661,206). (**a**) 65–74 years; (**b**) 75–84 years; and (**c**) ≥85 years. The medications analysed were based on the 2015 version of the American Geriatric Society’s Beers criteria. The differences in prescription rates between the age groups were statistically significant (*P*<0.001).
